# Racial disparity in utilization of therapeutic modalities among multiple myeloma patients: a SEER‐medicare analysis

**DOI:** 10.1002/cam4.1246

**Published:** 2017-11-03

**Authors:** Sikander Ailawadhi, Ryan D. Frank, Pooja Advani, Abhisek Swaika, M'hamed Temkit, Richa Menghani, Mayank Sharma, Zahara Meghji, Shumail Paulus, Nandita Khera, Shahrukh K. Hashmi, Aneel Paulus, Tanya S. Kakar, David O. Hodge, Dorin T. Colibaseanu, Michael R. Vizzini, Vivek Roy, Gerardo Colon‐Otero, Asher A. Chanan‐Khan

**Affiliations:** ^1^ Division of Hematology and Medical Oncology Mayo Clinic Jacksonville Florida; ^2^ Division of Biomedical Statistics and Informatics Mayo Clinic Rochester Minnesota; ^3^ Division of Biomedical Statistics Mayo Clinic Scottsdale Arizona; ^4^ Division of Hematology and Medical Oncology Mayo Clinic Scottsdale Arizona; ^5^ Division of Hematology Mayo Clinic Rochester Minnesota; ^6^ Colon and Rectal Surgery Mayo Clinic Jacksonville Florida; ^7^ Florida Administration Mayo Clinic Jacksonville Florida

**Keywords:** Cancer management

## Abstract

Outcomes have improved considerably in multiple myeloma (MM), but disparities among racial‐ethnic groups exist. Differences in utilization of novel therapeutics are likely contributing factors. We explored such differences from the SEER‐Medicare database. A utilization analysis of lenalidomide, thalidomide, bortezomib, and stem cell transplant (SCT) was performed for patients diagnosed with MM between 2007 and 2009, including use over time, use by race, time‐dependent trends for each racial subgroup, and survival analysis. A total of 5338 MM patients were included with median 2.4‐year follow‐up. Within the first year of MM diagnosis, utilization of lenalidomide, bortezomib, SCT, and more than one novel agent increased over time while utilization of thalidomide decreased. There was significantly lower utilization of lenalidomide among African‐Americans (*P *<* *0.01), higher thalidomide use among Hispanics and Asians (*P *<* *0.01), and lower bortezomib use among Asians (*P *<* *0.01). Hispanics had the highest median number of days to first dose of bortezomib (*P *=* *0.02) and the lowest utilization of SCT (*P *<* *0.01). Hispanics and Asians were the only groups without notable increases in lenalidomide and bortezomib use, respectively. SCT utilization increased over time for all except African‐Americans. SCT use within the first year after diagnosis was associated with better overall survival (HR 0.52; 95% CI: 0.4–0.68), while bortezomib use was associated with inferior survival (HR 1.14; 95% CI 1.02–1.28). We noted considerable variability in MM therapeutics utilization with seeming inequity for racial‐ethnic minorities. These trends should be considered to eliminate drug access and utilization disparities and achieve equitable benefit of therapeutic advances across all races.

## Introduction

Multiple myeloma (MM) is the second most common hematologic malignancy with approximately 30,000 new cases diagnosed in 2016 1. Death rates have been falling over the last decade with a reported 5‐year relative survival rate of 48.5% 1, 2, 3, 4. Despite these encouraging trends, disparities exist in outcomes by patient race‐ethnicity 3. Majority of the reported studies of racial disparities in survival have focused on White and African‐American patients 5, 6, 7, 8, 9. With changing United States (US) population demographics, investigations show that the disparities span fast growing minorities of Hispanics and Asians as well 3. While the causes of these outcome disparities by race may be multifactorial, access to and utilization of novel therapeutic agents and stem cell transplant (SCT) are likely noteworthy contributors 10, 11, 12, 13, 14, 15. Some studies have explored these disparities, although mostly focusing on Whites and African‐Americans 10, 12, 13, 16.

We performed an analysis of the utilization of novel therapeutic agents and SCT during the first year after diagnosis of MM across racial subgroups to better understand any patterns and explore any disparity that may help explain the differences in survival.

## Methods

### Data source

We utilized data from the National Cancer Institute's Surveillance, Epidemiology, and End Results (SEER) database with data linkage to Medicare claims (SEER‐Medicare, linkage completed in 2013) 17, 18. The Medicare files utilized included the Patient Enrollment and Diagnosis Summary file (PEDSF), the Outpatient file (institutional Medicare Part B claims), the Medicare Provider Analysis and Review file (inpatient Medicare Part A claims), the National Claims History file (provider Medicare Part B claims), Durable Medical Equipment files, and Medicare Part D file (prescription drug coverage for beneficiaries who purchase the benefit; approximately 60% of the beneficiaries) 19. The study received approval from the Institutional Review Board.

### Study population and variables

All cases of primary MM reported to the SEER cancer registry, defined by the *International Classification of Diseases*‐0‐3 code (version released September 18, 2015) of 9732 (MM) and microscopic confirmation from positive histology, cytology, or other microscopic method (diagnostic confirmation code of 1–4) were identified. Patients with any Medicare claims within 1 year after diagnosis of MM were included. Due to orally administered immunomodulatory drugs (IMiDs) being a therapeutic modality of interest and the availability of Medicare Part D data from 2007 onward only, incident cases with a confirmed diagnosis of MM between January 1, 2007 and December 31, 2009 with continuous Medicare coverage from 1 year prior to diagnosis through end of 2012 were included. Patients were divided into five mutually‐exclusive racial‐ethnic groups; non‐Hispanic White (White), non‐Hispanic African‐American (African‐American), non‐Hispanic Asian/Pacific Islander (Asian), Hispanic, and Native American (identified using the North American Association of Central Cancer Registries [NAACCR] Hispanic/Latino Algorithm [NAACCR Race & Ethnicity Work Group, 2011]) 20. Due to small numbers, Native Americans were not included in the statistical analyses. Patients with unknown race (0.6%) or race classified as other (0.4%) were excluded. Therapeutic modalities studied were lenalidomide, thalidomide, bortezomib, and SCT. Use of these modalities within the first 12 months of MM was obtained from the Medicare claims files using 5‐digit Healthcare Common Procedure Coding System (HCPCS) codes and 11‐digit National Drug Codes (NDC).

### Statistical analysis

Patient demographics and the use of lenalidomide, thalidomide, bortezomib, and SCT were summarized using frequencies/percent for categorical variables and median/interquartile ranges for continuous variables. Formal comparisons across race were performed using multivariate multinomial logistic regression with a glogit link, adjusted for the effects of age of diagnosis, year of diagnosis, and sex. Patients with any missing data were not included in analyses utilizing that covariate.

Multivariate associations (adjusted for the effects of age, race, and sex) between trends over time in any use, days of use, and days to first dose of bortezomib, lenalidomide, thalidomide, and SCT during the first year of MM were analyzed using a proportional odds model. A multivariate multinomial linear regression model was used to examine the association between therapeutic modality and race (adjusted for age, year, and sex). Associations between drug use by year were summarized separately for each race using a proportional odds model. Duration of follow‐up was defined as days from MM diagnosis to loss of follow‐up or death. Associations between drug use and time‐to‐death were assessed using a Cox proportional hazards model. A time‐dependent model was used to account for drug exposure varying over time. All analyses were performed using SAS version 9.4 (SAS Institute Inc.). All tests were two‐sided and *P *<* *0.05 was considered significant.

## Results

### Study cohort and demographics

**Figure 1:**
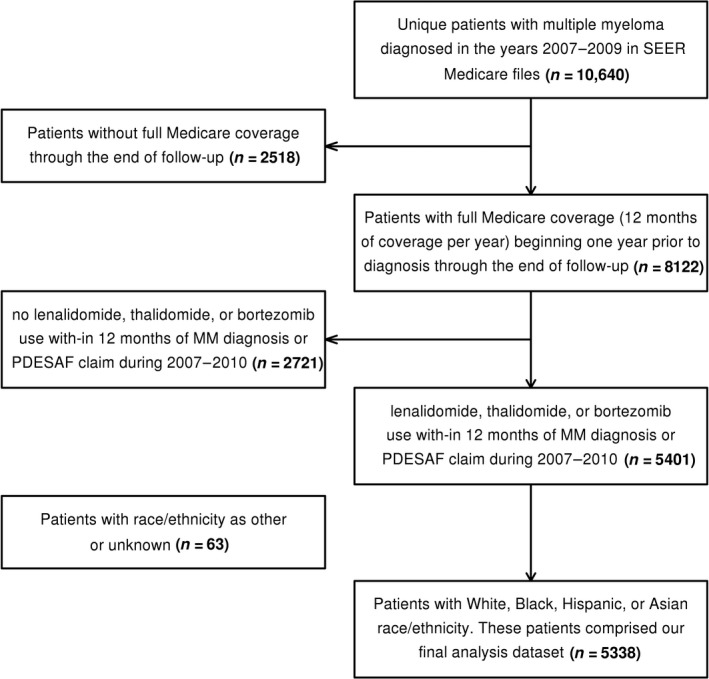
Explanation of patient counts that made up the final study cohort.

We identified 10,640 unique MM patients diagnosed within the specified time in the SEER‐Medicare files. Of these, 8122 patients had continuous Medicare coverage. We excluded patients with no lenalidomide, thalidomide, or bortezomib use within 12 months of MM diagnosis and those with no specific data on race‐ethnicity, leading to the final cohort of 5338 patients, of which 1680, 1793, and 1865 patients were diagnosed in years 2007, 2008 and 2009, respectively (Fig. 1). Patients included 2733 (51%) men and 2605 (49%) women with a median follow‐up of 2.4 years. Distribution by race included 3574 White (67%), 945 African‐American (17%), 531 Hispanic (10%), and 288 Asian (5%). Demographic characteristics at diagnosis were significantly different by race after adjusting for sex and year of diagnosis, with a median age of 74 years in African‐Americans (range 68–80) and Hispanics (range 70–80) versus 76 years in Whites (range 71–82) and Asians (range 71–81.5; *P *<* *0.01, Table 1). Sex distribution was significantly different among various racial subgroups after adjustment for year of diagnosis and age, with the highest proportion of males (53.4%) among Whites and the lowest proportion (45.2%) among African‐Americans (*P *<* *0.01, Table 1).

**Table 1:** Baseline characteristics of the multiple myeloma SEER cohort by patient race

Characteristic	White *N* = 3574	Hispanic *N* = 531	Black *N* = 945	Asian *N* = 288	Adjusted *P* value^a^
Age at diagnosis, median (Q1, Q3)^b^	76 (71, 82)	74 (70, 80)	74 (68, 80)	76.0 (71.0, 81.5)	<0.001
Year of diagnosis					0.920
2007	1123 (31.4%)	175 (33.0%)	287 (30.4%)	95 (33.0%)	
2008	1198 (33.5%)	180 (33.9%)	318 (33.7%)	97 (33.7%)	
2009	1253 (35.1%)	176 (33.1%)	340 (36.0%)	96 (33.3%)	
Male	1907 (53.4%)	259 (48.8%)	427 (45.2%)	140 (48.6%)	<0.001

a Overall Wald *P* value from a multinomial logistic regression model, after adjustment for sex, age at diagnosis and year of diagnosis.

b Q1: Quartile 1, Q3: Quartile 3.

### Therapeutic modality utilization during first year over time

**Figure 2:**
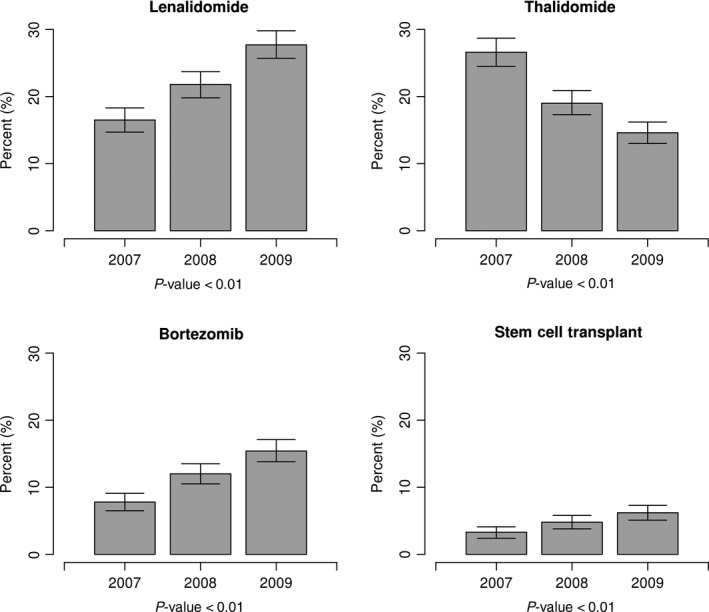
The use of various therapeutic modalities during first year of treatment over time (2007, 2008, and 2009) for multiple myeloma patients.

Lenalidomide use increased significantly from 2007 to 2009 (16.5–27.7%; *P *<* *0.01; Table 2, Fig. 2). Patients stayed on lenalidomide treatment for a longer duration, from median 84 days of use in 2007 to 112 days in 2009 (*P *<* *0.01), and started lenalidomide treatment sooner after diagnosis, with median days to first dose changing from 94 in 2007 to 65 in 2009 (*P *<* *0.01). For thalidomide, overall use decreased over time from 26.6% in 2007 to 14.6% in 2009 (*P *<* *0.01; Fig. 2). Median days of use for thalidomide decreased while median days to first dose increased slightly over time, but these trends were not statistically significant. Of all the novel therapeutic agents studied, bortezomib was used for the smallest proportion of patients with claims in the SEER‐Medicare database. Its use increased significantly over time from 7.8% in 2007 to 15.4% in 2009 (*P *<* *0.01; Fig. 2), and the median days to first dose of bortezomib decreased significantly from 67 in 2007 to 48 in 2009 (*P *=* *0.01). While SCT was utilized in a small proportion of Medicare‐eligible patients, its use increased significantly from 3.3% in 2007 to 6.2% in 2009 (*P *<* *0.01) over the studied time periods (Fig. 2). We also noted a significant increase in more than 1 novel agent use during the first year of MM diagnosis from 2.5% in 2007 to 5.7% in 2009 (*P *<* *0.01).

**Table 2:** Therapeutic modality utilization within first year of multiple myeloma diagnosis by year of diagnosis

Characteristic	2007 *n* = 1680	2008 *n* = 1793	2009 *n* = 1865	Adjusted *P* value^a^
Lenalidomide				
Any use, No. (%)	277 (16.5)	390 (21.8)	517 (27.7)	<0.01
Median days of use (Q1, Q3)^b^	84 (42, 147)	89 (42, 189)	112 (49, 196)	<0.01
Median days to first dose (Q1, Q3)	94 (34, 224)	79 (30, 191)	65 (30, 165)	0.02
Thalidomide				
Any use, No. (%)	447 (26.6)	342 (19.1)	272 (14.6)	<0.01
Median days of use (Q1, Q3)	118 (56, 224)	140 (56, 266)	112 (50, 224)	0.54
Median days to first dose (Q1, Q3)	36 (18, 83)	39 (20, 95)	40.5 (24, 102)	0.07
Bortezomib				
Any use, No. (%)	131 (7.8)	215 (12.0)	288 (15.4)	<0.01
Median days to first dose (Q1, Q3)	67 (38, 168)	50 (21, 123)	48 (21, 110)	0.01
Stem cell transplant				
Any use, No. (%)	55 (3.3)	86 (4.8)	116 (6.2)	<0.01
Use of 1 or more agents				<0.01
Bortezomib only, No. (%)	89 (5.3)	144 (8.0)	181 (9.7)	
Lenalidomide/thalidomide only, No. (%)	614 (36.5)	600 (33.5)	618 (33.1)	
Lenalidomide/thalidomide and bortezomib, No. (%)	42 (2.5)	71 (4.0)	107 (5.7)	

a Overall Wald *P* value from a Proportional odds model, after adjustment for sex, age at diagnosis, and patient race.

b Q1: Quartile 1, Q3: Quartile 3.

### Therapeutic modality utilization during first year by patient race

Associations between therapeutic modality utilization by race are presented in Table 3. Lenalidomide had the lowest utilization among African‐Americans (19.4%) and highest among Hispanics (23.7%; overall *P *<* *0.01). No significant difference was noted for median days of use, although they were lowest among Hispanics (84 days) and highest among Asians (112 days; overall *P *=* *0.72). Median days to first dose of lenalidomide after MM diagnosis was not statistically different by patient race (*P *=* *0.14), although it was only 67 days in African‐Americans compared to 139 days among Asians. Thalidomide use was significantly higher in Hispanics (27.9%) and Asians (28.5%) compared to Whites (18.3%) and African‐Americans (18.7%; overall *P *<* *0.01). Asians had the fewest median days of use for thalidomide (112 days) compared to all other races (140 days), but this difference was not statistically significant (overall *P *=* *0.56). Similarly, the difference in median days to first dose of thalidomide was not statistically significant by patient race (*P *=* *0.64). Bortezomib use was significantly different by race, being lowest among Asians (7.6%) and highest among Whites (12.8%; overall *P *<* *0.01). Hispanics had the highest median days from diagnosis to first dose of bortezomib (117 days), while the other groups had a significantly shorter period (median 46–51 days) (overall *P *=* *0.02). SCT use was significantly different by patient race, with 1.9% of Hispanic patients and 5.8% of White patients receiving it (overall *P *<* *0.01). We did not note a significant difference in time to SCT by patient race (*P *=* *0.49).

**Table 3:** Therapeutic modality utilization within first year of multiple myeloma diagnosis by patient race

Characteristic	White *n* = 3574	Hispanic *n* = 531	African‐American *n* = 945	Asian *n* = 288	Adjusted *P* value^a^
Lenalidomide					
Any use, No. (%)	814 (22.8)	126 (23.7)	183 (19.4)	61 (21.2)	<0.01
Median days of use (Q1, Q3)^b^	98 (49, 192)	84 (42, 172)	98 (42, 178)	112 (56, 189)	0.72
Median days to first dose (Q1, Q3)	79 (31, 186)	74 (29, 191)	67 (30, 165)	139 (35, 216)	0.14
Thalidomide					
Any use, No. (%)	654 (18.3)	148 (27.9)	177 (18.7)	82 (28.5)	<0.01
Median days of use (Q1, Q3)	140 (56, 224)	140 (56, 252)	140 (84, 252)	112 (56, 224)	0.56
Median days to first dose (Q1, Q3)	38 (20, 100)	43 (23, 84)	35 (18, 84)	38 (20, 61)	0.64
Bortezomib					
Any use, No. (%)	456 (12.8)	44 (8.3)	112 (11.9)	22 (7.6)	<0.01
Median days to first dose (Q1, Q3)	51 (23, 116)	117 (40, 212)	46 (22, 127)	50 (26, 140)	0.02
Stem cell transplant					
Any use, No. (%)	206 (5.8)	10 (1.9)	35 (3.7)	6 (2.1)	<0.01
Combination therapy					
Bortezomib only, No. (%)	301 (8.4)	22 (4.1)	80 (8.5)	11 (3.8)	<0.01
Lenalidomide/thalidomide only, No. (%)	1191 (33.3)	225 (42.4)	300 (31.7)	116 (40.3)	
Lenalidomide/thalidomide and bortezomib, No. (%)	155 (4.3)	22 (4.1)	32 (3.4)	11 (3.8)	

a Overall Wald *P* value from a multinomial logistic regression model, after adjustment for sex, age at diagnosis, and year of diagnosis.

b Q1: Quartile 1, Q3: Quartile 3.

**Figure 3:**
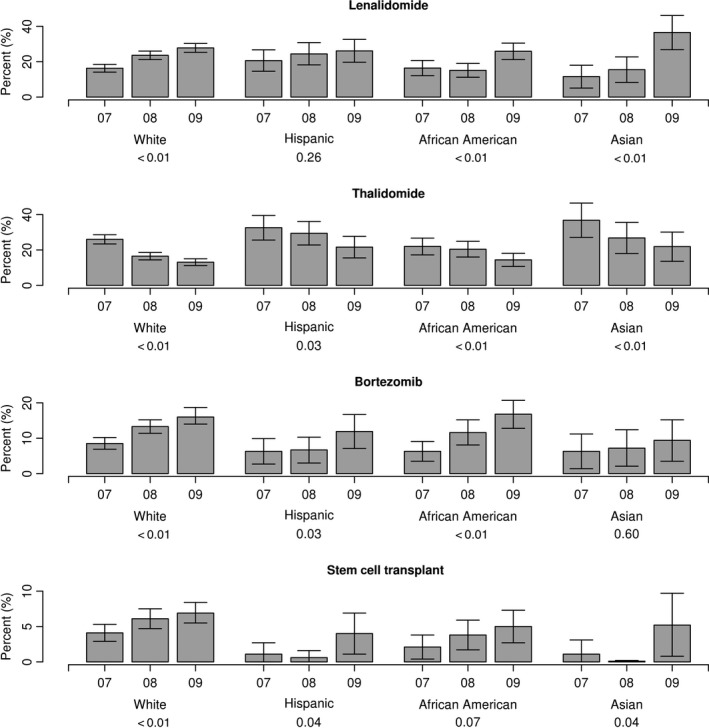
The use of various therapeutic modalities during first year of treatment over time (2007, 2008, and 2009) for multiple myeloma patients by race.

There were noteworthy trends in treatment utilization in the first year of MM diagnosis by race over time (Fig. 3). Lenalidomide use increased significantly from 2007 to 2009 for Whites (*P *<* *0.01), African‐Americans (*P *<* *0.01), and Asians (*P *<* *0.01), but not for Hispanics (*P *=* *0.26). To determine if the trends in lenalidomide use were significantly different by race, we ran a final multivariate model with a race‐by‐year interaction included. The *P*‐value was marginally significant (overall *P *=* *0.06), suggesting the trends in Hispanics were borderline significantly different than the other races. Over the same time period, thalidomide use significantly decreased for all racial subgroups. A significant increase in bortezomib use was noted over time for Whites (*P *<* *0.01), Hispanics (*P *=* *0.03), and African‐Americans (*P *<* *0.01), but not for Asians (*P *=* *0.60). We found a significant increase in SCT utilization over time for Whites (*P *<* *0.01), Hispanics (*P *=* *0.04), and Asians (*P *=* *0.04), but only a marginally significant trend in African‐Americans (*P *=* *0.07).

### Utilization of treatment modalities during first year and survival

Multivariate survival analysis showed an ordinal effect of worsening survival for every decade increase in patient age (hazard ratio [HR], 1.71; 95% CI, 1.63–1.79; *P *<* *0.01; Table 4). An inferior survival was noted for males (HR, 1.13; 95% CI, 1.06–1.21; *P *<* *0.01). No significant difference in survival was noted by patient race (*P *=* *0.10), or by use of lenalidomide (*P *=* *0.38) or thalidomide (*P *=* *0.30) but bortezomib use within the first year was associated with an inferior survival (HR, 1.14; 95% CI, 1.02–1.28; *P *=* *0.02), while use of SCT during first year was associated with better survival (HR, 0.52; 95% CI, 0.4–0.68; *P *<* *0.01).

**Table 4:** Associations with overall survival and therapeutic modality utilization within first year of multiple myeloma diagnosis using cox proportional hazards

Characteristic	Hazard ratio (95% CI)	Multivariate *P* value^a^
Age at diagnosis (per 10 years)	1.71 (1.63, 1.79)	<0.001
Race (ref White)		0.103
Hispanic	1.08 (0.97, 1.22)	
Black	1.11 (1.01, 1.22)	
Asian	1.06 (0.91, 1.23)	
Male (ref Female)	1.13 (1.06, 1.21)	<0.001
Lenalidomide use (ref no exposure)	0.96 (0.88, 1.05)	0.383
Thalidomide use (ref no exposure)	1.05 (0.96, 1.15)	0.302
Bortezomib use (ref no exposure)	1.14 (1.02, 1.28)	0.024
Stem cell transplant (ref no exposure)	0.52 (0.40, 0.68)	<0.001

a Overall Wald *P*‐value from a multivariate Cox proportional hazards regression. *P* value is adjusted for all characteristics listed in the table.

## Discussion

Introduction of novel treatments including proteasome inhibitors, IMiDs, and SCT have tremendously improved patient outcomes in MM 2, 4, 11, 12. It is recommended to utilize these options earlier in the management paradigm for better outcomes as the disease typically becomes refractory later on 21, 22, 23. While outcome disparities by race and ethnicity exist, potential inequities in the utilization of novel therapeutic approaches among MM patient subgroups need to be explored. We analyzed the comprehensive SEER‐Medicare database to explore these potential disparities and to understand the trends in utilization over time to see if they are becoming more equitable.

Medicare status for every individual is assessed continuously, and the coverage can change from month‐to‐month. We only included patients who had continuous Medicare coverage from 1 year prior to the diagnosis of MM to the end of the follow‐up period to avoid lapses in data due to intermittent coverage. Defined exclusions still yielded 5338 patients for the planned analyses. While this is a significant reduction in the number of patients included in the study's final analysis, such attrition has been seen in previous publications from the SEER‐Medicare database and are inherent to the nuances of data available through it 24. We noted that only a small proportion of patients with MM had claims data for lenalidomide, thalidomide, or bortezomib in our cohort, despite these agents being utilized almost universally for all MM patients. Medicare does not capture supplemental insurance utilization, and so drug claims in this database are lower than otherwise expected. Other groups have also reported similar rates of novel agent utilization from SEER‐Medicare 16. Of note, the study cohort in our analysis was defined by Medicare coverage and utilization and not disease epidemiology or treatment utilization other than Medicare.

Consistent with the current treatment guidelines, there was an increase in the utilization of lenalidomide and bortezomib and a decrease in thalidomide use within the first year of MM diagnosis over time. Furthermore, the median number of days to first treatment with lenalidomide or bortezomib decreased appreciably from 2007 to 2009, suggesting their earlier use in the treatment of MM patients and a longer duration of treatment within the first 12 months after diagnosis. We also noted an increase in exposure to both proteasome inhibitors and IMiDs during the first year of MM diagnosis. While we were not able to confirm concurrent use of these agents, it may be a possibility considering increasing acceptability of combination regimens 25. Of note, we report exposure to individual therapeutic agents and not the utilization of specific regimens, which are difficult to analyze from this database. Previous reports have shown an overall increase in SCT utilization across all MM patients over time and have outlined strategies to incorporate SCT in the management of elderly patients 12, 22, 26, but none of them have reported on Medicare‐eligible patients only. We noted a small, but meaningful increase in the utilization of SCT during the first year of MM diagnosis in our cohort, suggesting increased acceptance and feasibility even for Medicare‐eligible patients.

While all these trends over time were concordant with expected practice, we noted important differences in utilization by race. For IMiDs, Hispanics had the highest utilization of lenalidomide, while thalidomide use was substantially higher in Hispanics and Asians. African‐Americans had the lowest utilization of lenalidomide. Once the patients began receiving treatment with these agents, the duration of therapy was not appreciably different across races, although Hispanics and Asians had the lowest median duration for lenalidomide and thalidomide use, respectively. We also noted that Asians had the longest time to initial treatment with lenalidomide. A previous report has noted later initiation of novel therapeutics in African‐Americans compared with Whites 16. Our report is the first to look at differences in drug utilization in a cohort that is more representative of the current US population. We noted significantly different trends in bortezomib utilization, with the highest utilization among Whites and the longest median time to initial therapy among Hispanics. Early MM therapy with bortezomib‐based regimens is now frequently used, and such disparity in its utilization may explain the differences in outcomes reported previously for Hispanics 3. Another recent report has shown lower bortezomib utilization among African‐Americans but did not evaluate Hispanics separately 24. Previous reports have shown a lower utilization of SCT for African‐Americans compared to Whites and one recent report from the Center for International Blood and Marrow Transplant Research (CIBMTR) showed lowest rate of SCT utilization among Hispanics 12, 16, 24, 27. We confirmed these findings among Medicare‐eligible patients. A previous analysis reported that Whites and African‐Americans get treated with novel agent monotherapy at similar rates, but access to novel antimyeloma agent combinations is less frequent for African‐Americans 16. We did not evaluate concurrent utilization of novel agents, but noted that patients with bortezomib‐only utilization during the first year of MM diagnosis were more likely to be Whites or African‐American, while those with IMiD‐only utilization were more likely to be Hispanic or Asian. In addition to differences in access to various agents, treatment choices can differ based on disease presentation and comorbidities (e.g., preference of bortezomib‐based therapy in patients with renal dysfunction) which may differ by race 28. We attempted controlling for median income, education level and geographic region in our analysis but noted that since the sample size in some of the subgroups, especially racial minorities, was smaller, attempting to control for all these various sociodemographic factors made the sample size very heterogeneous with difficult to interpret results. All these identified sociodemographic factors are indeed extremely important and need to be analyzed in a larger data set with longer follow‐up since any comprehensive evaluation of novel, oral medications in MM would have to be limited to patients since 2007 when Medicare part D data became available. While we were not able to evaluate the impact of socioeconomic status on treatment utilization, limiting the analysis to the Medicare‐eligible population, with claims made only to Medicare rather than secondary insurances, probably minimizes such an impact.

Trends in the management of MM in the US have shown an increase in use of lenalidomide and bortezomib, with a decrease in thalidomide use over time 29. We noted that while overall these trends were true for our cohort as well, Hispanics did not have a meaningful increase in lenalidomide use and Asians did not have an increase in bortezomib use. These trends, are concerning as they suggest a lack of appropriate utilization of antimyeloma agents for Hispanics and Asians, the two fastest growing racial minority populations in the US. As noted previously, African‐Americans have had less access to SCT than Whites 12, 16. In our analysis, all racial groups other than African‐Americans saw a noteworthy increase in SCT use over time.

Survival analysis in our cohort did not show any differences by race, which is different from previous analyses including one from our group utilizing the SEER database 3. This may be due to a shorter follow‐up in the current analysis. As for the specific agents, we did not note a difference in survival by use of IMiDs in the first year, but patients who were treated with bortezomib only or who received both bortezomib and an IMiD during the first year of MM diagnosis had an inferior survival. In general, IMiDs have been the preferred agents for MM treatment in older patients 22, and bortezomib may be more often utilized for patients with aggressive disease, including those with poor‐risk cytogenetics or renal dysfunction 30, 31, 32, 33. Thus, the finding of inferior survival associated with bortezomib use during the first year of MM diagnosis may be a sign of selection bias, inherent to SEER‐Medicare analyses, as the database does not provide clinical parameters to corroborate this hypothesis. Nevertheless, so far there is no published report so far showing that risk‐stratification or the resulting choice of treatment due to it is different by patient race in MM. We noted an improvement in survival associated with the use of SCT during the first year of MM diagnosis, and while this may be reflective of the known benefit of SCT in MM, there may also be some selection bias since patients who are offered SCT, irrespective of their age, are more likely to be of better performance status with fewer comorbidities.

We present a comprehensive analysis of various novel therapeutic agents utilized for the management of MM during the first year after diagnosis and note considerable variability among patients of different racial‐ethnic subgroups. Treatment paradigms have been shifting rapidly in MM, nevertheless, agents such as bortezomib and lenalidomide as well as SCT remain cornerstones of patient management and their utilization is critical to patient outcomes. Our findings need to be examined in a larger cohort with comprehensive claims data, such as those available from commercial payers, to eliminate drug access and utilization disparities and achieve equitable benefit of therapeutic advances across all racial‐ethnic subgroups.

## Conflict of Interest

None declared.
